# Working with men to prevent intimate partner violence in a conflict-affected setting: a pilot cluster randomized controlled trial in rural Côte d’Ivoire

**DOI:** 10.1186/1471-2458-14-339

**Published:** 2014-04-10

**Authors:** Mazeda Hossain, Cathy Zimmerman, Ligia Kiss, Tanya Abramsky, Drissa Kone, Monika Bakayoko-Topolska, Jeannie Annan, Heidi Lehmann, Charlotte Watts

**Affiliations:** 1 Gender Violence & Health Centre, Department of Global Health & Development, London School of Hygiene & Tropical Medicine, London, UK; 2 The International Rescue Committee (IRC), New York, USA; 3 The International Rescue Committee (IRC), Abidjan, Côte d’Ivoire

**Keywords:** Conflict, Impact evaluation, Violence prevention, Men, Intimate partner violence

## Abstract

**Background:**

Evidence from armed conflict settings points to high levels of intimate partner violence (IPV) against women. Current knowledge on how to prevent IPV is limited—especially within war-affected settings. To inform prevention programming on gender-based violence in settings affected by conflict, we evaluated the impact of adding a targeted men’s intervention to a community-based prevention programme in Côte d’Ivoire.

**Methods:**

We conducted a two-armed, non-blinded cluster randomized trial in Côte d’Ivoire among 12 pair-matched communities spanning government-controlled, UN buffer, and rebel–controlled zones. The intervention communities received a 16-week IPV prevention intervention using a men’s discussion group format. All communities received community-based prevention programmes. Baseline data were collected from couples in September 2010 (pre-intervention) and follow-up in March 2012 (one year post-intervention). The primary trial outcome was women’s reported experiences of physical and/or sexual IPV in the last 12 months. We also assessed men’s reported intention to use physical IPV, attitudes towards sexual IPV, use of hostility and conflict management skills, and participation in gendered household tasks. An adjusted cluster-level intention to treat analysis was used to compare outcomes between intervention and control communities at follow-up.

**Results:**

At follow-up, reported levels of physical and/or sexual IPV in the intervention arm had decreased compared to the control arm (ARR 0.52, 95% CI 0.18-1.51, not significant). Men participating in the intervention reported decreased intentions to use physical IPV (ARR 0.83, 95% CI 0.66-1.06) and improved attitudes toward sexual IPV (ARR 1.21, 95% CI 0.77-1.91). Significant differences were found between men in the intervention and control arms’ reported ability to control their hostility and manage conflict (ARR 1.3, 95% CI 1.06-1.58), and participation in gendered household tasks (ARR 2.47, 95% CI 1.24-4.90).

**Conclusions:**

This trial points to the value of adding interventions working with men alongside community activities to reduce levels of IPV in conflict-affected settings. The intervention significantly influenced men’s reported behaviours related to hostility and conflict management and gender equitable behaviours. The decreased mean level of IPV and the differences between intervention and control arms, while not statistically significant, suggest that IPV in conflict-affected areas can be reduced through concerted efforts to include men directly in violence prevention programming. A larger-scale trial is needed to replicate these findings and further understand the mechanisms of change.

**Trial registration:**

clinicaltrials.gov NCT01803932

## Background

International awareness of violence against women and its impact on health and development has grown significantly over the past decade [1-3]. Violence against women includes sexual and physical violence perpetrated by non-partners and intimate partners. Research from around the world suggests that intimate partner violence (IPV) is widespread and that women bear the main burden. Globally, 30% of women aged 15 and over have reported physical and/or sexual violence from an intimate partner during their lifetime [4]. Violence against women, including physical and sexual abuse, coercion and threats, has been well-recognised as a public health issue with negative short and long-term physical health consequences (such as injuries, functional disorders, chronic pain and reproductive and sexual health problems), mental health consequences (including depression, anxiety, suicidal ideation and post-traumatic stress), and intergenerational and societal implications (including increasing healthcare costs, child abuse and homicide) [2,5-11].

To date, in sub-Saharan African conflict-affected settings, sexual violence has received considerable attention from the media and in reconstruction policy-making. However, at the same time, emerging prevalence data suggest that violence against women includes a much wider range of abuses than sexual violence alone, as levels of interpersonal violence or partner violence are extremely high in these settings. For example, in the Democratic Republic of Congo, a 2007 national survey found that more than half of ever-partnered women (56.9%) reported being physically assaulted by an intimate partner, 20% reported physical violence from a parent or in-law and 2.2% reported physical violence from police or soldiers. Sexual assault by an intimate partner was reported by 35.3% of women surveyed and 16% reported sexual violence from any perpetrator which included conflict-related sexual violence [12].

Interventions to address violence against women have traditionally focused on responses that provide assistance to survivors, such as shelter, legal advice and psychological support. Yet, recently, prevention has received growing attention for its potential to stop the violence before it starts, thereby potentially having a farther-reaching impact on reducing the prevalence of violence. Multi-faceted prevention programming has been developed to target risk factors associated with women’s experiences of intimate partner violence (such as alcohol abuse, young age, attitudes supportive of wife beating, multiple sexual partners outside marriage, experiences of childhood abuse and witnessing IPV as a child) [13,14]. Emerging evidence from non-conflict settings suggests that men’s normative attitudes (e.g., acceptability of wife beating) may be predictive of the perpetration of partner violence [14]. One current approach to prevention programming involves ‘gender-transformative’ strategies, where programmes target gender inequitable normative beliefs and behaviours that condone or encourage violence against women. To put this strategy into action, several violence prevention programmes have implemented male-focused interventions alongside programmes for women, with the aim of confronting gender norms related to negative manifestations of masculinity [15-18]. Evidence is beginning to show that by encouraging gender-equitable behaviours and beliefs it is possible to reduce men’s perpetration of intimate partner violence against women [19-27]. However, nearly all of this emerging evidence on prevention interventions has primarily been drawn from non-conflict affected settings, with little rigorous evidence on interventions that work directly with men in conflict-affected settings [14,28].

In 2010, the International Rescue Committee (IRC), a humanitarian aid agency that addresses gender-based violence (GBV) in humanitarian crisis settings through programmes for violence survivors, implemented a male-targeted violence against women prevention intervention using a gender transformative approach in Côte d’Ivoire. As part of the evaluation of this programme, a cross-sectional formative survey among communities receiving IRC programming was conducted in 2008. Results showed that 50% of ever-partnered women had experienced physical and/or sexual IPV in their lifetime and 84% of men agreed with the statement: ‘a woman should obey her husband even if she disagrees’ and nearly half of men (47%) affirmed at least one reason when it was acceptable for a man to hit his wife [29,30]. Building on earlier intervention work in Liberia and these research findings, a male-focused violence against women prevention intervention was developed for Côte d’Ivoire in October 2010. The aim of the programme was to shift gender norms and notions of masculinity that condone violence against women [31].

Côte d’Ivoire is a West African setting that has faced a protracted conflict known as *the Crisis. The Crisis* began with a coup d’état in 1999 and was punctuated with periods of active armed conflict-related violence until a temporary peace agreement was reached in 2007. Violent clashes re-emerged between 2009 and 2011 [32-34]. Following the election of a new president and government in 2012, the country has been undergoing a transition period from active conflict to stable peace building [34,35]. Côte d’Ivoire, once considered the ‘jewel of West Africa’, continues to remain a critical country for regional West African security as it maintains deep ties to neighbouring countries (Mali, Burkina Faso, Ghana, Guinea, Liberia) and other West African nations (Togo, Benin, Sierra Leone, Niger) through migration, trade and remittances. Little is currently known about the impact of over a decade of instability and violence on interpersonal violence [29,36]. Research conducted in Côte d’Ivoire may provide insights for the development and evaluation of violence prevention programming in conflict-affected settings.

Using a prospective cluster randomized controlled trial design, this pilot study aimed to assess the added value of a male-focused intervention to prevent intimate partner violence against women in communities that were receiving on-going community-level gender based violence prevention and response programming.

## Methods

### Overview

The evaluation of a complex intervention requires an approach that not only allows for the assessment of the intervention outcomes but also captures contextual factors and the implementation process. To this end, we used a prospective cluster randomized trial (CRT) design to evaluate the intervention among men in rural sites in Côte d’Ivoire. The CRT design used a mixed-methods approach (qualitative results reported separately) among pair-matched communities, where one community from each pair was randomly selected to be an intervention community and the other allocated as a control community. This type of design aims to compare outcomes between groups that receive and did not receive the proposed intervention. The study design included a baseline survey prior to the start of the intervention and a follow-up survey carried out one year after activities had ended. The primary trial analysis compared outcomes between the intervention and control communities at follow-up. The CRT design permitted the assessment of differences in gendered norms and behaviours and levels of IPV between intervention and control communities at follow-up, while controlling for any differences in these measures at baseline.

The trial objectives were to assess whether the Men’s Discussion Groups had an impact on the following outcomes: (1) women’s experiences of physical and/or sexual IPV in the last 12 months; (2) men’s reports of intention to use physical IPV; (3) men’s attitudes towards sexual IPV; (4) men’s use of hostility and conflict management skills; and (5) men’s participation in gendered household tasks.

### Intervention background: Men’s Discussion Group intervention and community-level programming

The intervention, ‘Men & Women in Partnership Initiative’, was developed to influence inequitable gendered attitudes, behaviours and expectations among men—with the ultimate aim of reducing intimate partner violence. The initiative centred on creating *Men’s Discussion Groups* using a 16-session curriculum designed to reduce overall levels of partner violence by:

•Increasing men’s knowledge about the impact of gender based violence on women, men and children;

•Shifting gender inequitable beliefs and behaviours around violence and household roles; and

•Providing men with hostility and conflict management skills as part of developing and sustaining new behaviours [37].

**Figure 1:**
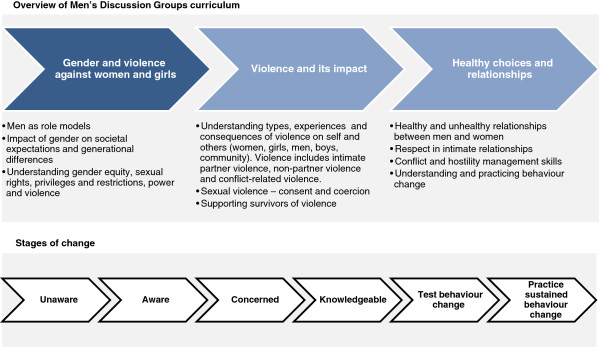
Overview of Men’s Discussion Groups curriculum and underlying stages of change.

By engaging men on a weekly basis over the course of four months, the Men’s Discussion Groups aimed to shift men’s attitudes from basic awareness about the impact and consequences of violence against women and girls to practicing and trialling behaviour change. The Men’s Discussion Groups offered participating men the opportunity to reflect on new attitudes and practice new behaviours within a supportive environment and to encourage social change within an intimate relationship. The curriculum draws upon social norm theory, which predicts that individuals behave in a manner that conforms to what they perceive to be normative behaviour. Therefore, the curriculum sought to challenge harmful normative attitudes and behaviours within the community and encourage positive male behaviours that participants could identify with and emulate in their own lives. Figure 1 presents an overview of the intervention components and stages of change [37].

Recruitment was open to all male community members (15+ years old) living in the study community and limited to 30 men per site. Participation was voluntary and no incentives were offered to participants. A lottery was used to select participants in communities where more than 30 eligible men volunteered. The Men’s Discussion Group goals were presented as separate but complementary to existing IRC community GBV response and prevention programming. All study sites had similar community-level programming to raise awareness about women’s rights and the consequences of GBV, and to create a GBV committee trained in basic support skills for violence survivors. The Men’s Discussion Group curriculum was developed by the IRC in 2010 to address the gap in prevention programming that worked directly with men. All intervention facilitators received a six-week multi-staged training. The final stage of the training included a facilitator-led pilot test after which the curriculum was modified to reflect suggested adaptations. The revised curriculum was then implemented within the study sites selected for intervention activities. Since the original piloting in Côte d’Ivoire in 2010, the Men’s Discussion Group intervention has become part of a larger IRC intervention package - Engaging Men in Accountable Practice (EMAP) – which has been implemented in other humanitarian crisis settings.

### Evaluation: sampling frame & eligibility

Twelve study sites (villages) across government-controlled, UN buffer, and rebel–controlled zones were identified from six administrative districts with established IRC community GBV response and prevention programming. Within each district two villages, matched on population size and socio-demographics were selected. Logistical challenges (e.g., accessibility) were also considered. Villages within each administrative district were separated by the presence of a geographical buffer (i.e., no direct routes between matched communities, no shared market centres) to avoid contamination of the control community via contact with intervention community members. Within each matched pair, one village was chosen at random and designated as the intervention site. During the Men’s Discussion Group intervention implementation, the control communities continued to receive the standard community GBV programming package and the intervention communities received the community GBV programming plus the Men’s Discussion Group intervention. At baseline (2010) and follow-up surveys (2012), all male intervention participants and their current female partners were interviewed. In the control communities, we selected male controls with exposure to community GBV programming via friendships with community members involved in GBV prevention activities. The men in the control arm were group age-matched to men in the intervention village pair and interviewed. All current female partners of the men in the control arm were also interviewed.

The sample size was determined by the number of participants in the Men’s Discussion Groups. Men’s Discussion Groups were created in six communities and limited to 30 men who volunteered to participate following an open community recruitment. Actual enrolment in the intervention varied slightly between communities. Men were followed up between time periods (baseline and one-year post intervention) and all female partners at the time of each interview were included in the analysis. The intervention could only be implemented within six communities due to limited human and financial resources. Given the small number of intervention clusters (six), our capacity to conduct statistical hypothesis tests was limited, therefore we present the unbiased impact estimates and associated confidence intervals, and discuss both the statistical significance of the results, including the direction and strength of the effects, and the broader plausibility of the findings.

### Outcome variables

The trial had five outcomes that were chosen prior to the follow-up survey to reflect the different aims of the Men’s Discussion Group intervention and the hypothesized theory of change [37]. Each outcome was generated as a binary variable:

*Women’s experience of physical and/or sexual intimate partner violence from a male partner in the past 12 months (women reporting).* Women’s past year exposure to physical and/or sexual IPV was measured using questions about experiencing specific acts of violence, the time period it occurred (last 12 months, before the last 12 months), and its frequency (once, a few times, often, never). Physical violence was considered to have occurred if an individual reported experiencing more than one physical violence act (slapped/pushed, hit with something that could hurt you) or at least one severe act of physical violence (kicked/dragged/beaten, choked/burned, or threatened with a weapon) from their current intimate partner. Sexual violence was recorded when at least one act of forced or coerced sex was reported. Women’s reports of physical and/or sexual IPV in the past year were considered the primary outcome. As part of the secondary analysis the impact of the intervention on levels of physical IPV and sexual IPV were assessed separately.

Women’s reports of IPV experiences were used to estimate levels of male perpetration of IPV against a female partner rather than relying on men’s self-reports of IPV perpetration. The decision to use women’s reports was based on our previous experience conducting surveys relating to IPV against women where we found men underreported perpetration in relation to women’s reports of experiencing violence. This pattern was confirmed using data from the community formative research [29] and baseline survey of this trial, [30] as well as in other recent studies conducted by our research centre and others [38,39]. In addition, we hypothesized that bias in male reporting (towards underreporting) would likely become more extreme after contact with a violence prevention intervention, thereby potentially inflating effect estimates and making the intervention appear more effective at reducing violence than it actually had on participants. By using female reports of IPV experiences, we are therefore providing more conservative (and realistic) estimates of intervention effect.

*Intention to use physical IPV (men reporting).* Individual intentions to commit physical IPV against a female partner were measured. At follow-up, a man’s intention to commit a physically violent act was measured using an 8-item series of questions to assess whether he would hit his partner *right now* in response to particular situations, such as if ‘she tried to control him’, ‘she came home late’, or ‘she nagged him’. Men who agreed with at least one situation where he would hit his wife were coded as holding beliefs to use physical IPV. These measures were adapted from the Proximal Antecedents to Violent Episodes (PAVE) scale [40].

*Attitudes towards sexual IPV: Wife can refuse sex (men reporting).* We assessed normative beliefs around sexual IPV (women can refuse sex from her male partner) using items developed for the WHO Multi-Country Study on Domestic Violence [1]. Men were asked if it was acceptable for a woman to refuse sex with her husband given the following situations: 1) she does not want to have sex; 2) he is drunk; 3) she is sick; 4) he mistreats her; 5) she suspects he has been unfaithful; 6) she knows that he has been unfaithful; and 7) he refuses to use condoms. Men who agreed with all seven statements were coded as holding improved attitudes towards sexual IPV.

*Use of hostility and conflict management skills (men reporting and women reporting no threats from male partner).* A list of positive and negative reactions men might have when angry was used to identify men who used positive hostility and conflict management skills. Men were also given an opportunity to provide options not on the pre-defined skills list and these free text responses were categorized. Men’s reports were used as the primary data source, however, to reduce bias only men whose female partners did not report being threatened were coded as having used a positive conflict management technique. The list of skills was developed using feedback from Men’s Discussion Group facilitators on techniques discussed during the intervention.

*Male involvement in household tasks typically done by females (men reporting).* Men were asked who (you/shared equally/your partner/others) took care of the following tasks: preparing meals, washing dishes, sweeping/cleaning the house, washing clothes, fetching water or bathing the children. Men were considered to be involved in a household task traditionally done by women (e.g., washing dishes, sweeping) if they reported contributing to two or more tasks in the past 12 months either by themselves or sharing the task equally.

### Control variables

In the adjusted multivariate models, we controlled for the following: age groups (15–24; 25–34; 35–49; 50–85 years); cohabitation status (individuals sharing same household, binary); ability to read (considered a potential confounder influencing men’s participation, binary) and traumatic experiences (domains common among war-affected populations) and baseline levels of the outcomes. Questions from the Harvard Trauma Questionnaire were used to capture experiences potentially associated with conflict-related violence [41,42]. All participants were asked if and when they had ever experienced a traumatic event within domains generally applicable to traumatised populations. The domains included: war-like conditions, bodily injury, forced confinement and coercion (e.g. forced to engage in sex for food or protection), harmed others, disappearance/death/injury of loved one, threats against you or loved ones, and ‘afraid for life’ [41]. A score of one point was assigned for each time point at which an experience was reported within each domain, overall scores thus took into account both frequency/duration and breadth of trauma experienced. A binary variable was created with exposure categories 0–4 and ≥5, using the median score as the cut-off point for the more highly exposed group.

### Study instrument

Our questionnaire [43] drew from violence and health outcome modules including the WHO Multi-country Study on Women’s Health & Domestic Violence against Women [1], the LSHTM violence and health among women asylum seekers study [5], and a trial on intimate partner violence and HIV prevention in Uganda [38]. All men and women were asked the same questions. The questionnaire was developed in English and French and translated and back-translated into eight local Ivorian languages using an intensive group translation process developed during the field worker training. All field staff translated the French version of the questionnaire individually into a local language and then met with other field staff in a language group to reach a consensus on the translation and to account for variations in dialects. The translated measures were then compared with the seven other language groups and the lead researcher to ensure that local language and French versions shared equivalent meanings and were culturally appropriate [29]. At the final phase of the training, the questionnaire was piloted and revised accordingly.

### Training & ethical procedures

Data was collected in French or one of eight local languages and no interpreters were used. Interviewers received an intensive 2.5 weeks of training, which included sensitive interviewing techniques for trauma-exposed populations, and gender and violence awareness sessions. Political instability, risk of renewed violence and vulnerability of both the study population and the field workers necessitated the development of strict ethical and safety procedures [44]. Procedures were developed to prioritize the security and well-being of participants and the field workers, minimize and respond to psychological distress, ensure available referral and support options and included multiple follow-up safety inquiries during and after fieldwork [45].

Notably, couples in intimate partnerships were interviewed at baseline and follow-up. For safety reasons, in most settings it is not recommended that interviews about IPV are conducted among couples and that research teams with little experience of conducting research on violence deviate from these established safety guidelines [45]. In this study, a team experienced in GBV research worked closely with the intervention team to establish a multi-staged information and inquiry process (before, during and after the data collection) to ensure the safety of all participants and field staff. Only couples that were involved in the Men’s Discussion Group or familiar with GBV community awareness activities were interviewed. All participants had access to appropriate follow-up referrals if requested. And, to ensure that the research process was transparent, multiple discussions with household leaders and male partners were held to inform them about the aim of the research. Individuals therefore would not fear taking part in the private face-to-face interviews as community support was built before any data collection activities were undertaken. Continuous monitoring was also conducted by the research and intervention teams. Participant informed consent was obtained before starting any interviews and this was accompanied by ‘stop’ procedures so trained field staff would cease data collection within a community if they perceived an individual’s participation might be potentially harmful to themselves or others. The research supervisors followed up with any inquiries and referral requests that arose during interviews. These additional precautions were implemented to ensure that no harm resulted from being involved in the study. Follow-up checks after data collection by the intervention team found no adverse effects from participation in the research.

Ethical and safety approval was received from the Ethics Committee at the London School of Hygiene & Tropical Medicine. Local ethical approval was received from the Ministry of Family, Women and Social Affairs in Côte d’Ivoire in 2008.

### Primary and secondary analysis

We had limited power to detect statistically significant intervention effects (as determined by the convention of reporting significance where the p-value is less than 0.05) due to the small cluster numbers. Therefore, the trial was designed to yield unbiased measures of effect. Our findings focus on the direction, consistency and coherence of observed results, as well as an assessment of the statistical significance of the outcome indicators. We conducted a cluster-level analysis comparing outcomes among intervention males (and their female partners) and age-matched male controls (and their female partners) to measure intervention impact. The approach we use follows the basic principles for the analysis of cluster randomized trials as set out by Donner and Klar [46] and is similar to that used in several recent studies evaluating community-based HIV and violence prevention interventions in Africa [21,47,48].

The analysis was done on an *Intention to Treat* (ITT) basis, whereby respondents were analyzed according to community assignment (regardless of whether men attended sessions). This was done to account for any diffusion that may have occurred among the men who knew each other. For each outcome, site-level past year prevalence was generated. The geometric mean of site-level prevalence was calculated for intervention and control villages respectively, and the ratio of these two figures (geometric mean prevalence ratio) at follow-up was used to give an estimate of crude intervention effect. An unpaired *t*-test was then used to compare the logarithms of the prevalence figures and assess the statistical significance of the difference in outcomes between intervention and control sites [21,46,47].

The generation of adjusted risk ratios (ARR) for each of the outcome measures involved two stages. First an individual-level binary logistic regression model, in which the dependent variable is the binary outcome of interest, was fitted to data from control villages. Independent variables in this model included potential confounders decided upon *a prior* (age, cohabitation status, self-reported literacy, above median level of exposure to war-related traumatic events, and a baseline prevalence measure of the respective outcome indicator). This model was used to predict the number of people in each site (intervention and control sites) that would be expected to experience the outcome at follow-up in the absence of the intervention. For each site, the ratio of observed to expected numbers with the outcome was then calculated (O/E). A geometric mean of these site-level summary measures was calculated for intervention and control sites respectively, and a ratio of these means was used to generate a point estimate of the adjusted intervention effect. As with the crude estimates, an unpaired *t*-test was used to assess the statistical significance of this comparison and construct 95% CIs around the adjusted risk ratio.

To assess the effect of active participation in the Men’s Discussion Group intervention (rather than the effect of enrolment in the intervention), a secondary analysis was performed at the cluster-level. We included in the village-level summaries only men who were active participants of the Men’s Discussion Group intervention as indicated by their attendance of 13 sessions or more (intervention arm) and a sample of age-matched men in control communities (control arm). The number of sessions was chosen based on the intervention course content. It was anticipated that by 13 sessions, all of the expected intervention outcomes would have been addressed.

In all cases, the direction of intervention effects on the outcomes was interpreted on the basis of the magnitude of adjusted risk ratios. Risk ratios of 1.1 or greater were considered to indicate an increase in the outcome associated with the intervention, those of 0.9 or less to indicate a decrease in the outcome, while those closer to 1 were interpreted as indicative of no association. We hypothesized at the start of the evaluation, the direction of intervention effect on each of the primary outcomes (Table 1).

**Table 1:** Hypothesized direction of intervention effect on outcomes

**OUTCOMES (comparison between intervention and control arms)**		**Hypothesized DIRECTION OF EFFECT**
***Intervention outcome***	**Research outcomes**	
*Levels of male IPV perpetration*	- Past year perpetration of physical and/or sexual IPV	DECREASE
	- Past year perpetration of physical IPV	DECREASE
	- Past year perpetration of sexual IPV	DECREASE
*Intention and attitudes towards IPV (physical and sexual violence)*	- Intention to use physical violence against an intimate partner (in at least one circumstance)	DECREASE
	- Attitudes: Believes a woman can refuse sex in any circumstance	INCREASE
*Use of hostility & conflict management skills*	- Positive hostility and conflict management techniques used in last 12 months and none of his female partners report him threatening her during arguments	INCREASE
*Shift in roles and behaviours towards gender equity in relationships and gender norms*	- Man involved in at least two household tasks during last 12 months	INCREASE

## Results

### Participation levels

**Figure 2:**
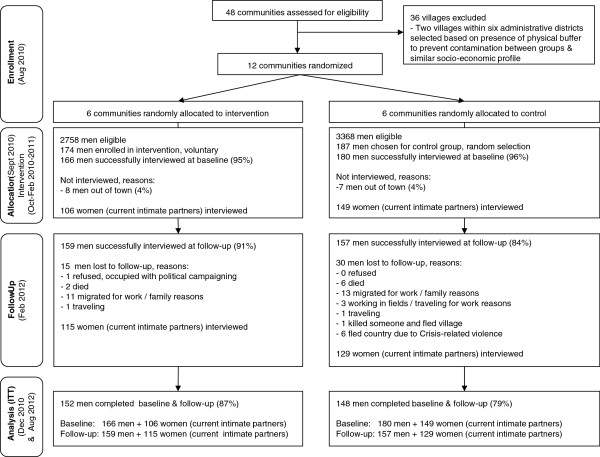
Flow diagram.

Among the 174 men who enrolled in the intervention cohort, 166 (95%) men completed the baseline interviews and 159 (91%) completed the follow-up interview. In control communities, 180 (96%) men completed interviews at baseline and 157 (84%) completed the follow-up. (Figure 2) Men’s Discussion Group sessions had attendance levels of 50% or higher for most individual sessions. Among men enrolled in the intervention (across all six intervention sites), 52% attended 13 or more of the 16 sessions.

Similar proportions of women completed interviews at baseline and follow-up. Among females with a male partner who participated in a Men’s Discussion Group, 103 completed baseline and follow-up interviews, 17 completed the baseline interview only and 24 completed the endline interview only. Within the control communities, 129 women completed both, 33 completed baseline only and 23 completed the endline only. All current female partners at endline were interviewed. Female partners at baseline were not necessarily the same at endline for various reasons including divorce, death, or migration due to the conflict while some men had married second or third wives between baseline and follow-up (Figure 2).

### Study participants

Baseline characteristics of study participants are presented in Table 2. Men in the intervention and control communities shared similar characteristics with more variation between women. Most participants lived with their current intimate partner (>78%) and 11% of men in intervention and control communities reported being in a polygamous relationship. The mean age of men in intervention and control communities was 40 years old. The majority of men earned an income through agriculture (75% intervention, 76% control), while women reported income through agriculture (36% intervention, 34% control) and small business activities (47% intervention, 59% control). Variations were noted in literacy levels, which were higher among men in the intervention arm (76%), compared to those in the control arm (68%). Traumatic experiences were also slightly higher among intervention communities (45% men, 38% women) than control communities (35% men, 29% women) at baseline.

**Table 2:** Demographic characteristics

**Baseline characteristics**	**Intervention villages**		**Control villages**	
	**Men n (%)**	**Women n (%)**	**Men n (%)**	**Women n (%)**
***Household-level***				
**Household has electricity**	74/166 (45%)	53/106 (53%)	64/180 (36%)	79/149 (53%)
**Main source of drinking water is tap/piped (private or public)**	30/166 (18%)	15/106 (14%)	34/180 (19%)	23/149 (15%)
**Household has a mobile phone**	122/166 (73%)	74/106 (70%)	122/180 (68%)	108/149 (72%)
***Individual-level***				
**Age (years)**	Mean = 40.0, sd = 11.6	Mean = 34.2, sd = 10.0	Mean = 39.6, sd = 13.6	Mean = 32.1, sd = 11.3
**Main ethnic/language groups**				
*Baoulé*	32/166 (19%)	17/106 (16%)	57/180 (32%)	46/149 (31%)
*Gueré*	43/166 (26%)	21/106 (20%)	16/180 (9%)	14/149 (9%)
*Yacouba*	28/166 (17%)	26/106 (25%)	41/180 (23%)	44/149 (30%)
*Beté*	1/166 (1%)	1/106 (1%)	27/180 (15%)	20/149 (13%)
*Gouro*	27/166 (16%)	15/106 (14%)	5/180 (3%)	3/149 (2%)
*Niamboua*	16/166 (10%)	7/106 (7%)	0 (0%)	0 (0%)
*Mossi*	6/166 (4%)	4/106 (4%)	11/180 (6%)	5/149 (3%)
*Wobé*	3/166 (2%)	7/106 (7%)	1/180 (1%)	1/149 (1%)
*Dioula/Malinké*	2/166 (1%)	1/106 (1%)	2/180 (1%)	2/149 (1%)
*Senoufo*	2/166 (1%)	3/106 (3%)	1/180 (1%)	1/149 (1%)
**Religion**				
*Christian*	67/166 (40%)	63/106 (59%)	75/180 (42%)	103/149 (69%)
*Muslim*	18/166 (11%)	12/106 (11%)	24/180 (13%)	16/149 (11%)
*Animist*	61/166 (37%)	3/106 (3%)	49/180 (27%)	5/149 (3%)
*No religion*	16/166 (10%)	27/106 (25%)	27/180 (15%)	21/149 (14%)
**Lived in study village as a child (<12 yrs old)**	84/166 (51%)	18/106 (17%)	82/180 (46%)	43/149 (29%)
**Ever attended school**	140/166 (84%)	49/106 (46%)	123/178 (69%)	57/146 (39%)
**Able to read**	125/164 (76%)	32/103 (31%)	120/175 (68%)	46/147 (31%)
**Does not earn an income**	2/166 (1%)	17/106 (16%)	5/180 (3%)	13/149 (9%)
**Farmer/farm owner**	124/166 (75%)	38/106 (36%)	136/180 (76%)	51/149 (34%)
**Small business owner**	10/166 (6%)	50/106 (47%)	11/179 (6%)	88/149 (59%)
**Ever-partnered**	163/166 (98%)	106/106 (100%)	180/180 (100%)	149/149 (100%)
**Current partnership status**				
*No current partner*	16/166 (10%)	0 (0%)	4/180 (2%)	0 (0%)
*Currently living with partner*	129/166 (78%)	99/106 (93%)	159/180 (88%)	135/149 (91%)
*Currently with partner, not living together*	17/166 (10%)	7/106 (7%)	14/180 (8%)	14/149 (9%)
**Polygamous relationship**	18/167 (11%)	15/106 (14%)	20/180 (11%)	18/149 (12%)
Experienced traumatic events in at least 5 domains, or on at least 5 separate occasions*	72/159 (45%)	43/114 (38%)	55/155 (35%)	36/125 (29%)

*Domains included: war-like conditions (village attacked, forced to flee village, forced to hide in bush); bodily injury (seriously injured by weapon or fighting, beaten by armed forces, forced to have sex with someone who attacked village); forced confinement and coercion (forced to work for someone who attacked village, forced to engage in sex in exchange for something such as food or protection for family); harmed others (used gun or weapon against someone, seriously injured someone, forced to kill someone in self defence); disappearance/death/injury of loved one (member of family/someone close seriously injured or killed by violence); threats against you or loved ones (someone in family threatened, harassed by armed forces with threats to life); mental health (afraid for life).

### Impact on trial outcomes

The direction of effect for all of the trial outcomes was in the hypothesized directions. (Table 1) One year following the end of intervention activities, comparing the primary outcomes at follow-up between intervention and control communities we found a decrease in women’s experiences of physical and sexual IPV (ARR = 0.52, 95% CI 0.18 - 1.51), although this trend was not statistically significant. (Table 3) We also found a lower prevalence of men’s reported intention to commit physical IPV (ARR 0.83, 95% CI 0.66 - 1.06) and increased levels of men who believe a woman has the right to refuse sex under all circumstances (ARR 1.21, 95% CI 0.77 - 1.91). The trial found statistically significant impacts on men’s reported use of hostility and conflict management skills and men’s reported involvement in gendered household tasks. Specifically, men’s reported use of positive hostility and conflict management skills increased among men in intervention communities, who were significantly more likely to report using at least one positive conflict management technique compared to men in control communities (ARR 1.30, 95% CI 1.06 - 1.58). Similarly, men who had been part of the Men’s Discussion Group intervention were more likely to report having helped with gendered household chores than men in control communities (ARR 2.47, 95% CI 1.24 - 4.90) (Table 3).

**Table 3:** Multivariate secondary analysis of intervention effect on trial primary and secondary outcomes at follow-up

**Trial outcomes (secondary outcomes in italics)**	**Baseline**		**Follow-up**		**Unadjusted RR**^**¥**^**(95% CI)**	**Adjusted RR**^**¥**^**(95% CI)**
	**Intervention**	**Control**	**Intervention**	**Control**		
**Intimate Partner Violence (IPV)** (Women’s reports)						
Experience of physical and/or sexual IPV, last 12 months	26/106 (25%)	35/149 (23%)	13/113 (12%)	22/126 (17%)	0.57 (0.21 – 1.53)	0.52* (0.18 – 1.51)
*Experience of physical IPV, last 12 months*	*21/106 (20%)*	*22/149 (15%)*	*9/113 (8%)*	*9/126 (7%)*	*1.06 (0.39 – 2.86)*	0.64* (0.24 – 1.73)
*Experience of sexual IPV, last 12 months*	*12/106 (11%)*	*20/149 (13%)*	*7/112 (6%)*	*18/126 (14%)*	*0.51 (0.18 – 1.45)*	0.50* (0.14 – 1.80)
**Intention and Attitudes towards IPV** (Men’s reports)						
Intention to use physical violence against an intimate partner (in at least one circumstance)	n/a	n/a	64/159 (40%)	75/155 (48%)	0.81 (0.66 – 0.99)	0.83*^±^ (0.66 – 1.06)
Believes a woman can refuse sex in all circumstances	44/166 (27%)	43/180 (24%)	63/159 (40%)	56/155 (36%)	1.26 (0.83 – 1.91)	1.21* (0.77 – 1.91)
**Hostility & Conflict Management Skills** (Men’s report of skill, women’s reports of no threats)						
Man uses at least one hostility/conflict management technique and none of his female partners report him threatening her during arguments	n/a	n/a	130/159 (82%)	100/156 (64%)	1.26 (1.08 – 1.48)	1.30*^±^ (1.06 – 1.58)
**Male Involvement in Household** (Men’s reports)						
Man involved in at least two household tasks, last 12 months	69/149 (46%)	40/151 (26%)	76/142 (54%)	25/144 (17%)	4.04 (1.53 – 10.65)	2.47* (1.24 – 4.90)

^¥^Risk ratios calculated at the cluster-level using data from follow-up. Both crude and adjusted ratios were adjusted for village-pair and weighted according to the number of observations per village.

*Adjusted risk ratios generated on the basis of expected number of events from a logistic regression model on individual data with independent variables including man’s age, cohabitation status, self-reported ability to read, above median level of exposure to war-related traumatic events, and baseline measure of outcome indicator (or most similar baseline indicator available).

^±^Attitudes towards a man’s use of physical IPV against his wife, used as most similar baseline measure in calculation of adjusted risk ratio.

### Secondary analysis

We performed a secondary analysis to explore an intervention dose-effect among those attending more intervention sessions (at least 13). No greater effects were found among those who attended more sessions (compared to controls) versus those attending fewer (versus controls) except for physical IPV, which is borderline significant among men attending fewer than 13 sessions (Table 4).

**Table 4:** Multivariate analysis comparing high dose receivers (more than 13+ sessions) of Men’s Discussion Group intervention versus age-matched controls, and low dose receivers (12 or fewer sessions) versus age-matched controls

**Trial outcomes (secondary outcomes in italics)**	**Low dose vs. control (N = 71 men in each group, and 46 most recent female partners in each group)**		**High dose vs. control (N = 86 men in each group, and 67 most recent female partners in each group)**	
	**Unadjusted RR**^**¥**^**(95% CI)**	**Adjusted RR**^**¥**^**(95% CI)**	**Unadjusted RR**^**¥**^**(95% CI)**	**Adjusted RR**^**¥**^**(95% CI)**
**Intimate Partner Violence (IPV)** (Women’s reports)				
Experience of physical and/or sexual IPV, last 12 months	0.64 (0.22 – 1.89)	0.59* (0.18 – 1.90)	0.76 (0.38 – 1.55)	0.68* (0.31 – 1.49)
*Experience of physical IPV, last 12 months*	1.11 (0.46 – 2.68)	0.60* (0.39 – 0.94)	1.31 (0.63 – 2.71)	0.90* (0.31 – 2.66)
*Experience of sexual IPV, last 12 months*	0.64 (0.22 – 1.89)	0.70* (0.18 – 2.76)	0.68 (0.25 – 1.85)	0.61* (0.22 – 1.68)
**Intention and Attitudes towards IPV** (Men’s reports)				
Intention to use physical violence against an intimate partner (in at least one circumstance)	0.94 (0.80 – 1.11)	0.95*^±^ (0.71 – 1.27)	0.57 (0.31 – 1.05)	0.60*^±^ (0.33 – 1.06)
Believes a woman can refuse sex in all circumstances	1.39 (0.89 – 2.19)	1.33* (0.86 – 2.07)	1.08 (0.68 – 1.72)	1.03* (0.62 – 1.71)
**Hostility & Conflict Management Skills** (Men’s report of skill, women’s reports of no threats)				
Man uses at least one hostility/conflict management technique and none of his female partners report him threatening her during arguments, last 12 months	1.23 (1.01 – 1.49)	1.27*^±^ (1.00 – 1.60)	1.27 (1.07 – 1.51)	1.31*^±^ (1.04 – 1.64)
**Male Involvement in Household** (Men’s reports)				
Man involved in at least two household tasks, last 12 months	3.72 (2.32 – 5.95)	2.03* (1.44 – 2.87)	2.98 (0.88 – 10.03)	2.04* (0.86 – 4.83)

^¥^Risk ratios calculated at the cluster-level using data from follow-up. Both crude and adjusted ratios were adjusted for village-pair and weighted according to the number of observations per village.

*Adjusted risk ratios generated on the basis of expected number of events from a logistic regression model on individual data with independent variables including man’s age, cohabitation status, self-reported ability to read, above median level of exposure to war-related traumatic events, and baseline measure of outcome indicator (or most similar baseline indicator available).

^±^Attitudes towards a man’s use of physical IPV against his wife, used as most similar baseline measure in calculation of adjusted risk ratio.

Looking at the trends in violence over time, we found a downward trend in physical and/or sexual IPV in both intervention and control communities. The reductions in physical IPV between baseline and follow-up were slightly larger in intervention communities than in controls. Interestingly, while there did seem to be reductions in sexual violence between baseline and follow-up within intervention communities, there was no change between baseline and follow-up for sexual IPV within control communities. The belief that women can refuse sex from her partner increased in both intervention and control sites (although not significantly).

## Discussion and conclusions

The findings suggest that a short but focused intervention with men (Men’s Discussion Groups) can change men’s behaviour. In this trial we found a statistically significant increase in participants’ reported use of techniques to manage hostility and conflict, and involvement in household tasks among men in the intervention communities. Additionally, among these men, there was also a lower prevalence of physical and/or sexual IPV perpetration, lowered intention to use physical IPV and improved beliefs that women can refuse sex, although the differences were not statistically significant. The observed changes for all outcomes were in the hypothesized direction, which suggests that the changes seen are unlikely to be due to chance alone (e.g., in which case we would expect to see some outcomes increase and others decrease). Although the limited number of clusters (due to logistical and budgetary constraints) reduced our statistical power, the use of a prospective, cluster randomized design with age-matched controls in socio-demographically matched communities enabled us to produce unbiased effect estimates of the intervention outcomes.

To our knowledge, this study is among the first to present data from a cluster randomized trial on a male-focused IPV prevention intervention in a conflict-affected setting. Our evaluation measured the impact at one year post-intervention to capture more sustained changes. It is recognised that changing normative behaviours around IPV is not a rapid process and it is possible that this timeframe was too short to see a large effect. However, within this time frame, behaviours and gender-related attitudes (i.e., associated with IPV perpetration), such as increased participation in household tasks and conflict management skills, may have been more readily influenced than changes in the incidence of violence.

Although the intervention was not associated with a statistically significant reduction in men’s perpetration of physical and/or sexual intimate partner violence, this must be interpreted with caution, given the limited statistical power of the study. The findings suggest that the intervention contributed to a decrease in factors commonly associated with the perpetration and normalization of intimate partner violence [13,14,49,50]. In addition, the good attendance rates for most groups, significant increases in use of conflict management techniques, and significant increases in male involvement in household tasks typically done by females suggest that the intervention had an impact in shifting gender norms and notions of masculinity that condone violence against women.

The relative decrease in intimate partner violence within the intervention communities point to the potential added value of supplementing community-level GBV prevention programming (services for survivors, economic empowerment, advocacy, research and learning, and community mobilization) with male-targeted interventions such as the Men’s Discussion Groups. Men’s newly acquired skills to manage hostility and conflict may have enabled them to reduce the use of violence by providing them with techniques for moderating emotional reactions. It is not possible to discern if the trend towards lower levels of IPV is due solely to the Men’s Discussion Group intervention, as we also noted a decrease in physical IPV levels, albeit smaller, across the control sites. Nonetheless, it is important to note that physical violence levels in intervention communities were higher at baseline than in control communities, and at follow-up, violence levels were lower among intervention (20% at baseline to 8% at follow-up) versus control (15% at baseline to 7% at follow-up) communities. The relative decrease between baseline and follow-up levels of IPV between intervention and control communities suggests that the existing comprehensive community GBV programming package may also have an influence on the violence perpetrated by men exposed to the community programme, and that the Men’s Discussion Group intervention may have an added value of furthering this trend.

This study was subject to several limitations. Selection bias was possible, as men who chose to enrol in the intervention may have been open to the intervention content and thus more amenable to change. To address this potential bias, we selected male controls that had some exposure to GBV prevention activities through friendships with community members involved in community GBV activities. In addition, the secondary analysis based on attendance needs to be interpreted with caution as men who attended a higher number of sessions may not be comparable to all men in the control arm. There was also a strong potential for reporting bias, with men who have been exposed to the intervention being more likely to report positive programmatic effects. However, a particular strength of our study was our use of women’s reports of their partner’s behaviours, where theoretically justifiable (i.e., past year experiences of IPV). This process however, also necessitated that extra care and steps were taken prior to, and after, data collection to ensure that no one was harmed as a result of participating in the research.

In a study population affected by armed conflict, some loss to follow-up was inevitable due to situations such as insecurity related to conflict violence, migration due to fleeing an attack and natural migration due to change in marital status or work. We attempted to mitigate loss to follow-up by tracking down, whenever logistically feasible, individuals interviewed at baseline. This included sending field workers to other communities and in one case, organising the transportation of a couple that had re-located to a nearby refugee camp for a day to complete the follow-up interviews. These attempts proved successful and loss-to-follow-up was kept to a minimum in both intervention and control communities.

Importantly, these findings illustrate the potential impact of an intervention working with men. These results suggest that questions about working with men and potential mechanisms to facilitate broader societal change need to be further examined for future programme development. A larger-scale trial is needed to replicate these findings and further understand the mechanisms of change. Another strength of this trial is that despite the challenges inherent with working in a conflict setting, such as disruptions in communities due to violence and traumatised study populations, the findings illustrate that it is possible to conduct rigorous evaluations under challenging circumstances.

Violence against women in conflict-affected settings has emerged prominently on the international agenda [51]. Our study results come from a war-torn country and illustrate the potential impact of intervening with men to shift gender norms and the great potential to prevent violence against women—and within a very short programmatic period. As the international community coordinates with national governments to design and fund reconstruction programming to foster safe and healthy post-conflict communities, prevention activities that include women *and* men should be at the heart of these development strategies.

## Competing interests

The authors declare that they have no competing interests.

## Authors’ contributions

MH, CZ, CW were responsible for the design and conduct of the study. MH, DK, LK were responsible for training and piloting. MH, DK were responsible for all fieldwork. MBT was responsible for country-level coordination of intervention and research activities. MH, TA were responsible for statistical data analysis. MH, CZ, LK, CW, DK, MBT, JA, HL contributed to the interpretation of the data. MH, drafted the manuscript which was reviewed and approved by all authors. MH had full access to all of the data in the study and takes responsibility for the integrity of the data and the accuracy of the data analysis. All authors read and approved the final manuscript.

## Pre-publication history

The pre-publication history for this paper can be accessed here:

http://www.biomedcentral.com/1471-2458/14/339/prepub
